# Supplementary figures and images for: Antero-posterior patterning in the brittle star Amphipholis squamata and the evolution of echinoderm body plans
Source: EvoDevo. 2025 May 31;16:7. doi: 10.1186/s13227-025-00244-8 (PMC12126913; doi:10.1186/s13227-025-00244-8)

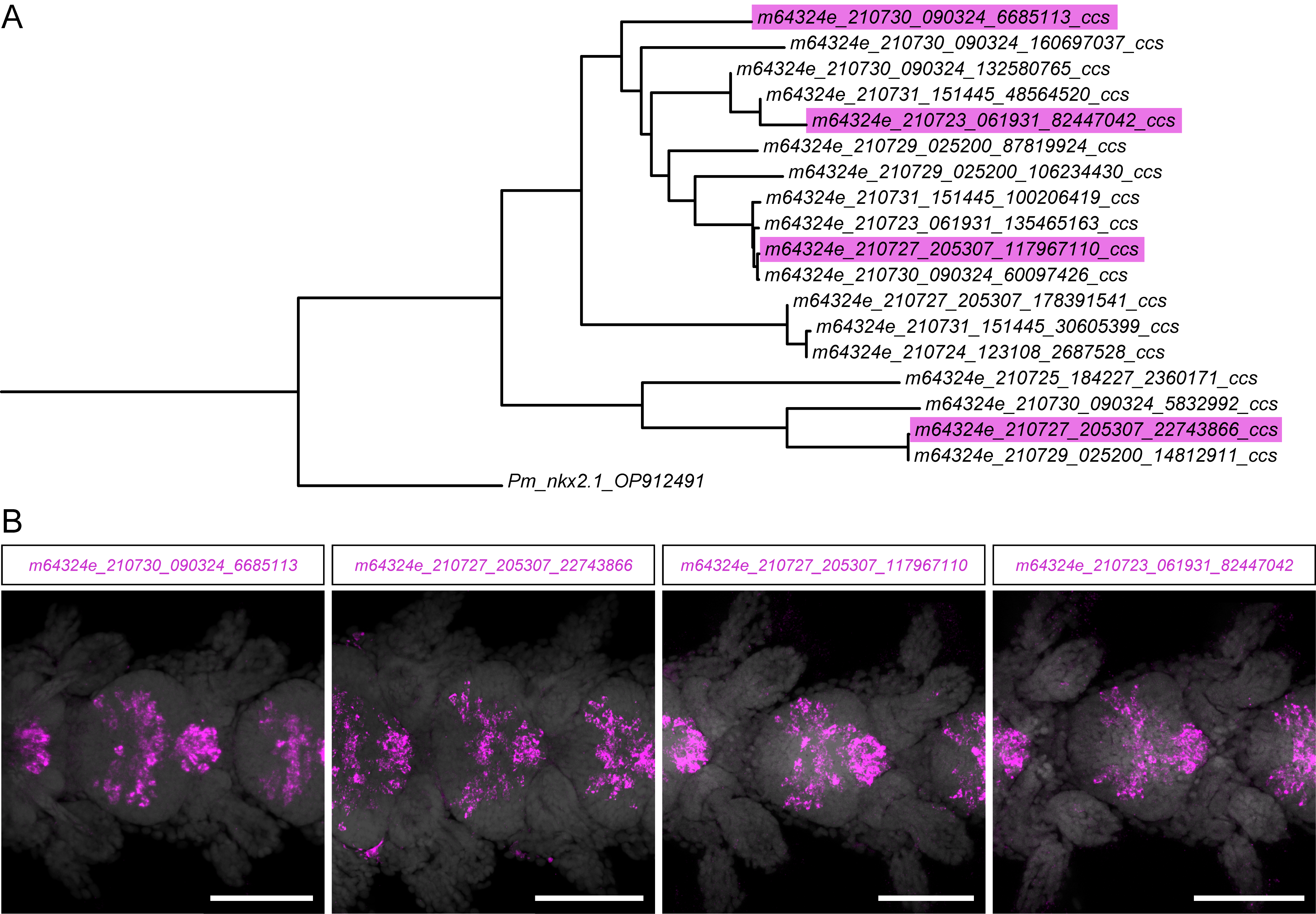

Supplement: Supplementary file 1 — Additional file 1: Fig. 1. Comparison of expression of nkx2.1 sequence variants. A, Neighbor-joining tree showing 18 different nkx2.1 sequence variants identified in Amphipholis squamata. The tree is rooted using Patiria miniata nkx2.1. B, single HCRs for four different nkx2.1 sequence variants showing detailed oral views of late juvenile arms. The sequence variants used for HCRs are highlighted in magenta in the tree in. All samples are counterstained with DAPIto mark cell nuclei. Scale bars: 100 µm. [file 13227_2025_244_MOESM1_ESM.tif]

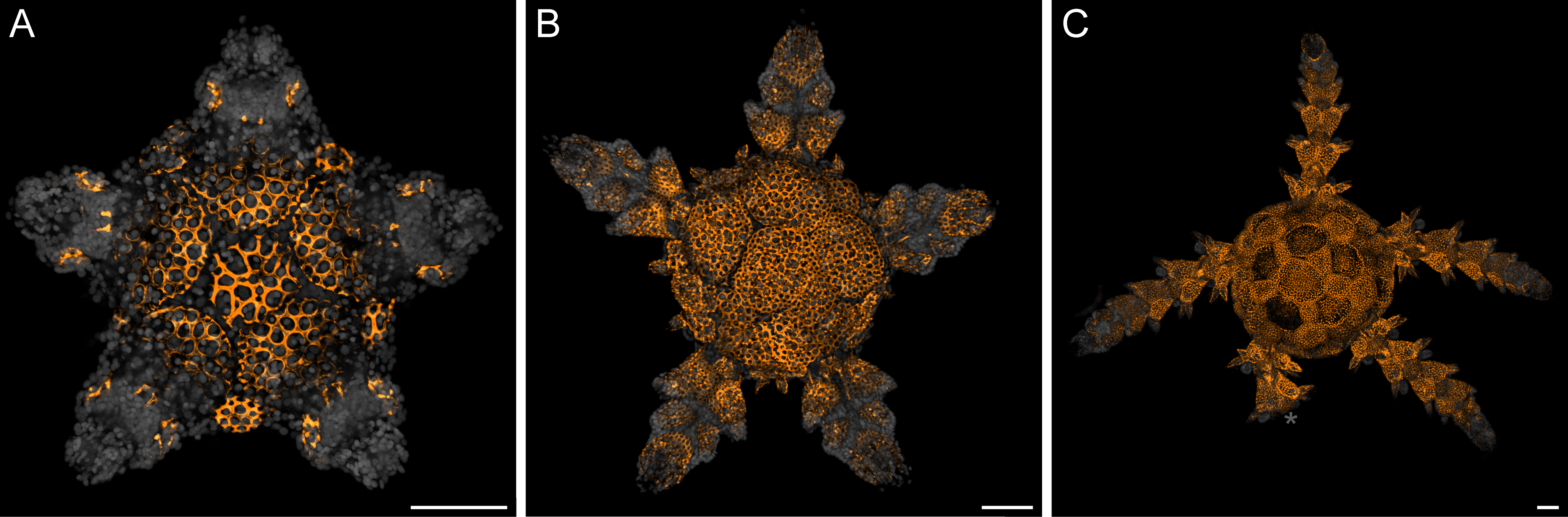

Supplement: Supplementary file 2 — Additional file 2: Fig. 2. Aboral views of Amphipholis squamata calcein stainings. Calcein stainingslabeling the endoskeleton of early, mid-and lateAmphipholis squamata whole-mount juveniles viewed from the aboral side. Note that the late juvenile has a damaged arm. All samples are counterstained with DAPIto mark cell nuclei. Scale bars: 100 µm. [file 13227_2025_244_MOESM2_ESM.tif]

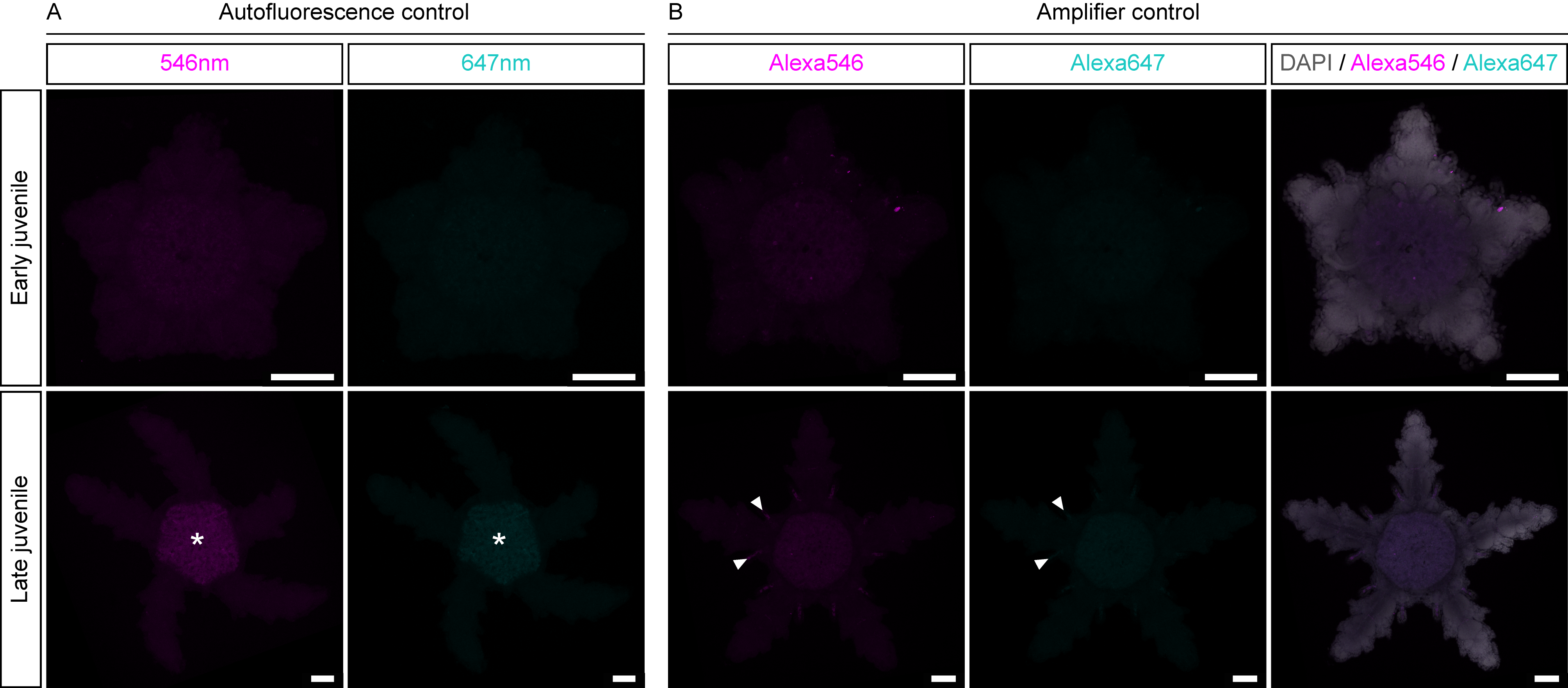

Supplement: Supplementary file 3 — Additional file 3: Fig. 3. HCR controls. A. Controls for background autofluorescence imaged at 546 nm and 647 nm in the absence of probes and amplifiers in Amphipholis squamata early and late whole-mouth juveniles. B. Controls for amplifier specificityimaged at 546 nm and 647 nm in the absence of probes in early and late whole-mouth juveniles. On the right panel, Alexa546 and Alexa647 amplifiers are shown as composite image of the same samples, counterstained with DAPIto mark cell nuclei. Note that in some samples, there are low levels of autofluorescence in the digestive tract, especially at later stages, and that amplifier trapping in the lumen of the tube feetmay be present. In both cases, aspecific staining can be accurately differentiated from specific HCR staining by its low intensity and perfect overlaps across the 546 nm and 647 nm acquisition channels. Scale bars: 100 µm. [file 13227_2025_244_MOESM3_ESM.tif]

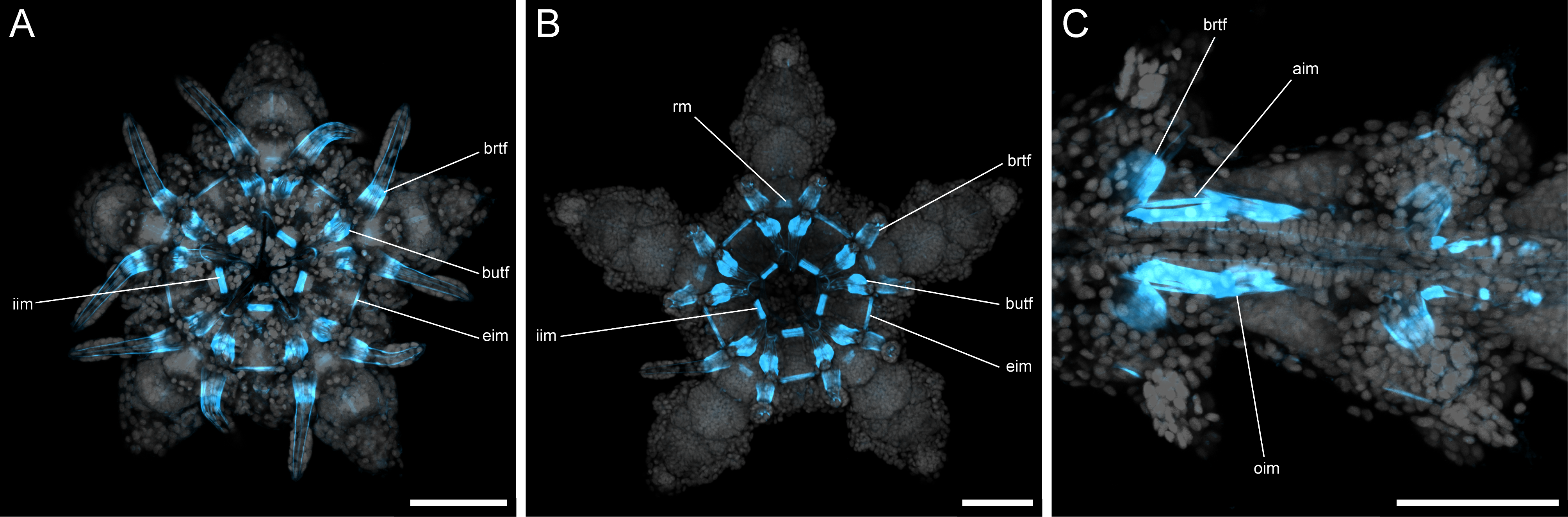

Supplement: Supplementary file 5 — Additional file 5: Fig. 5. Phalloidin stainings of Amphipholis squamata juveniles. Phalloidin stainings labelling F-actinof earlyand mid-Amphipholis squamata whole-mount juveniles viewed from the oral side and of a detailed oral viewof a brachial segment at the late juvenile stage. All samples are counterstained with DAPIto mark cell nuclei. aim: aboral intervertebral muscle, brtf: brachial tube foot, butf: buccal tube foot, eim: external interradial muscle, iim; internal interradial muscle, oim: oral intervertebral muscle, osp: rm: radial muscle. Scale bars: 100 µm. [file 13227_2025_244_MOESM5_ESM.tif]
