# Supplementary material for: Antero-posterior patterning in the brittle star Amphipholis squamata and the evolution of echinoderm body plans
Source: EvoDevo. 2025 May 31;16:7. doi: 10.1186/s13227-025-00244-8 (PMC12126913; doi:10.1186/s13227-025-00244-8)
Supplement: Supplementary file 4 — Additional file 4: Fig. 4. Phylogenetic trees of Amphipholis squamata orthologues. Phylogenetic relationship of Amphipholis squamata genes investigated in this study. A, Hox phylogeny. B, ANTP class homeobox transcription factors phylogeny. C, PRD class homeobox transcription factors phylogeny. D, Zic transcription factors phylogeny. E, SINE and TALE class homeobox transcription factors phylogeny. F, Myosin Heavy Chains phylogeny. G, Elav RNA binding proteins phylogeny. H, Hedgehog ligands phylogeny. I, Frizzled and secreted frizzled receptors phylogeny. Phylo­genetic trees are based on sequences from mouse, amphioxus, hemichordate, echinoids, asteroid, ophiuroidand polychaete. GenBank accession numbers are indicated between brackets. Trees were calculated using both Maximum Likelihoodand Bayesian Inferencemethods. Only the MLor BItrees are shown, with branch lengths being representative of sequence substitution rates, and branch support indicated as posterior probabilities from the BI analysis/bootstrap percentages from the ML analysis. “–” indicates that the branching patterns of the ML and BI analyses diverged at this node. [file 13227_2025_244_MOESM4_ESM.pdf]

A

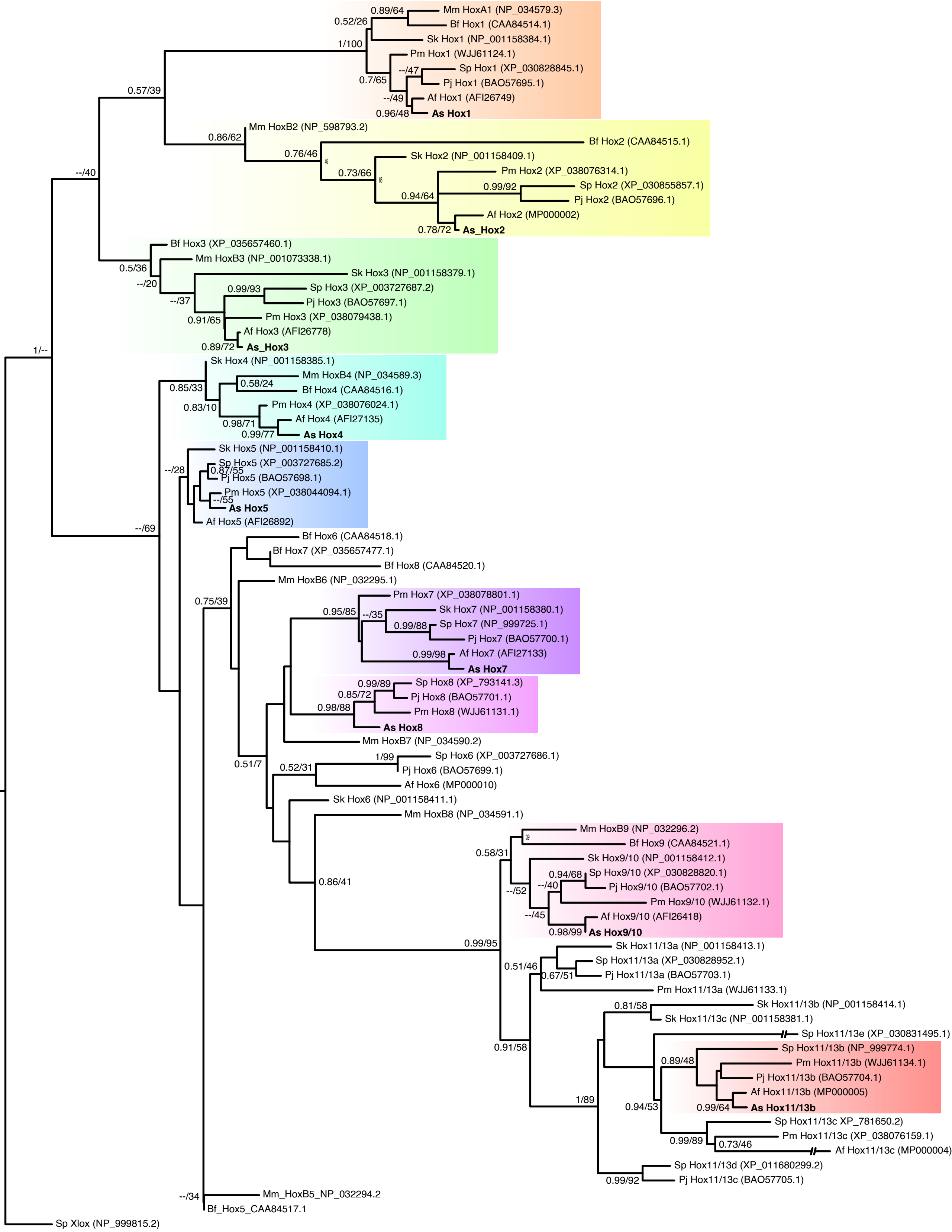

0.89/64
Mm HoxA1 (NP_034579.3)
0.52/26
Bf Hox1 (CAA84514.1)
Sk Hox1 (NP_001158384.1)
1/100
Pm Hox1 (WJJ61124.1)
--/47
Sp Hox1 (XP_030828845.1)
0.7/65
Pj Hox1 (BAO57695.1)
--/49
Af Hox1 (AFI26749)
0.96/48
As Hox1
0.57/39
Mm HoxB2 (NP_598793.2)
0.86/62
Bf Hox2 (CAA84515.1)
0.76/46
Sk Hox2 (NP_001158409.1)
0.73/66
Pm Hox2 (XP_038076314.1)
0.99/92
Sp Hox2 (XP_030855857.1)
0.94/64
Pj Hox2 (BAO57696.1)
Af Hox2 (MP000002)
0.78/72
As_Hox2
--/40
Bf Hox3 (XP_035657460.1)
Mm HoxB3 (NP_001073338.1)
0.5/36
--/20
Sk Hox3 (NP_001158379.1)
0.99/93
Sp Hox3 (XP_003727687.2)
--/37
Pj Hox3 (BAO57697.1)
Pm Hox3 (XP_038079438.1)
0.91/65
Af Hox3 (AFI26778)
0.89/72
As_Hox3
1/--
Sk Hox4 (NP_001158385.1)
Mm HoxB4 (NP_034589.3)
0.58/24
0.85/33
Bf Hox4 (CAA84516.1)
0.83/10
Pm Hox4 (XP_038076024.1)
0.98/71
Af Hox4 (AFI27135)
0.99/77
As Hox4
Sk Hox5 (NP_001158410.1)
Sp Hox5 (XP_003727685.2)
0.87/55
--/28
Pj Hox5 (BAO57698.1)
Pm Hox5 (XP_038044094.1)
--/55
As Hox5
Af Hox5 (AFI26892)
--/69
Bf Hox6 (CAA84518.1)
Bf Hox7 (XP_035657477.1)
Bf Hox8 (CAA84520.1)
Mm HoxB6 (NP_032295.1)
0.75/39
Pm Hox7 (XP_038078801.1)
0.95/85
--/35
Sk Hox7 (NP_001158380.1)
0.99/88
Sp Hox7 (NP_999725.1)
Pj Hox7 (BAO57700.1)
0.99/98
Af Hox7 (AFI27133)
As Hox7
0.99/89
Sp Hox8 (XP_793141.3)
0.85/72
Pj Hox8 (BAO57701.1)
0.98/88
Pm Hox8 (WJJ61131.1)
As Hox8
Mm HoxB7 (NP_034590.2)
1/99
Sp Hox6 (XP_003727686.1)
0.51/7
0.52/31
Pj Hox6 (BAO57699.1)
Af Hox6 (MP000010)
Sk Hox6 (NP_001158411.1)
Mm HoxB8 (NP_034591.1)
Mm HoxB9 (NP_032296.2)
Bf Hox9 (CAA84521.1)
0.58/31
Sk Hox9/10 (NP_001158412.1)
0.94/68
Sp Hox9/10 (XP_030828820.1)
0.86/41
--/40
Pj Hox9/10 (BAO57702.1)
--/52
Pm Hox9/10 (WJJ61132.1)
--/45
Af Hox9/10 (AFI26418)
0.98/99
As Hox9/10
0.99/95
Sk Hox11/13a (NP_001158413.1)
Sp Hox11/13a (XP_030828952.1)
0.51/46
0.67/51
Pj Hox11/13a (BAO57703.1)
Pm Hox11/13a (WJJ61133.1)
0.81/58
Sk Hox11/13b (NP_001158414.1)
Sk Hox11/13c (NP_001158381.1)
Sp Hox11/13e (XP_030831495.1)
0.91/58
Sp Hox11/13b (NP_999774.1)
0.89/48
Pm Hox11/13b (WJJ61134.1)
Pj Hox11/13b (BAO57704.1)
1/89
Af Hox11/13b (MP000005)
0.94/53
0.99/64
As Hox11/13b
Sp Hox11/13c XP_781650.2)
Pm Hox11/13c (XP_038076159.1)
0.99/89
0.73/46
Af Hox11/13c (MP000004)
Sp Hox11/13d (XP_011680299.2)
0.99/92
Pj Hox11/13c (BAO57705.1)
--/34
Mm_HoxB5_NP_032294.2
Bf_Hox5_CAA84517.1
Sp Xlox (NP_999815.2)

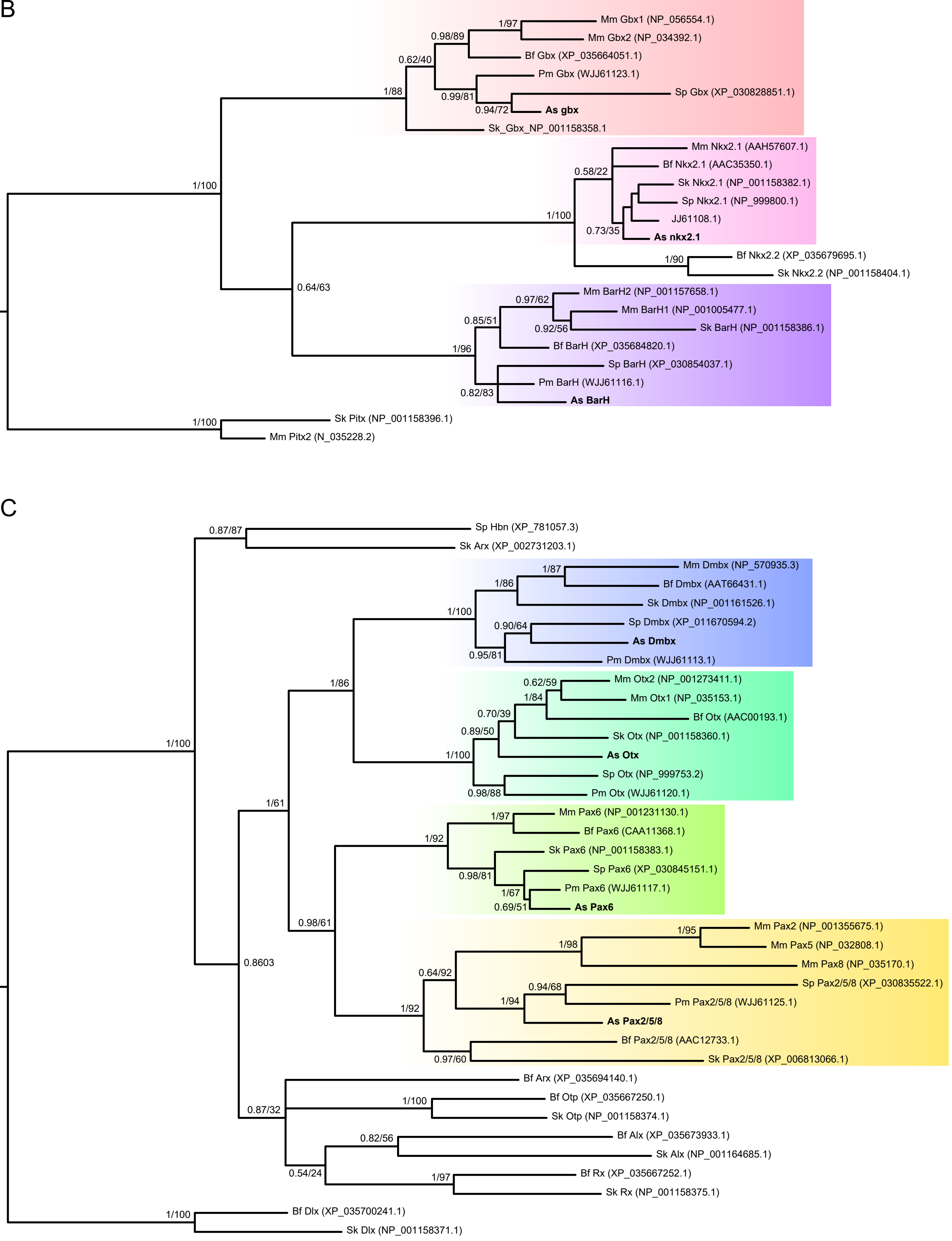
B
1/97
Mm Gbx1 (NP_056554.1)
0.98/89
Mm Gbx2 (NP_034392.1)
0.62/40
Bf Gbx (XP_035664051.1)
Pm Gbx (WJJ61123.1)
1/88
0.99/81
Sp Gbx (XP_030828851.1)
0.94/72
As gbx
Sk_Gbx_NP_001158358.1
Mm Nkx2.1 (AAH57607.1)
Bf Nkx2.1 (AAC35350.1)
0.58/22
1/100
Sk Nkx2.1 (NP_001158382.1)
Sp Nkx2.1 (NP_999800.1)
1/100
JJ61108.1)
0.73/35
As nkx2.1
1/90
Bf Nkx2.2 (XP_035679695.1)
Sk Nkx2.2 (NP_001158404.1)
0.64/63
Mm BarH2 (NP_001157658.1)
0.97/62
Mm BarH1 (NP_001005477.1)
0.85/51
0.92/56
Sk BarH (NP_001158386.1)
1/96
Bf BarH (XP_035684820.1)
Sp BarH (XP_030854037.1)
Pm BarH (WJJ61116.1)
0.82/83
As BarH
1/100
Sk Pitx (NP_001158396.1)
Mm Pitx2 (N_035228.2)
C
0.87/87
Sp Hbn (XP_781057.3)
Sk Arx (XP_002731203.1)
1/87
Mm Dmbx (NP_570935.3)
1/86
Bf Dmbx (AAT66431.1)
Sk Dmbx (NP_001161526.1)
1/100
0.90/64
Sp Dmbx (XP_011670594.2)
As Dmbx
0.95/81
Pm Dmbx (WJJ61113.1)
1/86
0.62/59
Mm Otx2 (NP_001273411.1)
1/84
Mm Otx1 (NP_035153.1)
0.70/39
Bf Otx (AAC00193.1)
0.89/50
Sk Otx (NP_001158360.1)
1/100
1/100
As Otx
Sp Otx (NP_999753.2)
0.98/88
Pm Otx (WJJ61120.1)
1/61
1/97
Mm Pax6 (NP_001231130.1)
Bf Pax6 (CAA11368.1)
1/92
Sk Pax6 (NP_001158383.1)
0.98/81
Sp Pax6 (XP_030845151.1)
1/67
Pm Pax6 (WJJ61117.1)
0.69/51
As Pax6
0.98/61
1/95
Mm Pax2 (NP_001355675.1)
1/98
Mm Pax5 (NP_032808.1)
0.8603
Mm Pax8 (NP_035170.1)
0.64/92
0.94/68
Sp Pax2/5/8 (XP_030835522.1)
1/94
Pm Pax2/5/8 (WJJ61125.1)
1/92
As Pax2/5/8
Bf Pax2/5/8 (AAC12733.1)
0.97/60
Sk Pax2/5/8 (XP_006813066.1)
Bf Arx (XP_035694140.1)
1/100
Bf Otp (XP_035667250.1)
0.87/32
Sk Otp (NP_001158374.1)
0.82/56
Bf Alx (XP_035673933.1)
Sk Alx (NP_001164685.1)
0.54/24
1/97
Bf Rx (XP_035667252.1)
Sk Rx (NP_001158375.1)
1/100
Bf Dlx (XP_035700241.1)
Sk Dlx (NP_001158371.1)

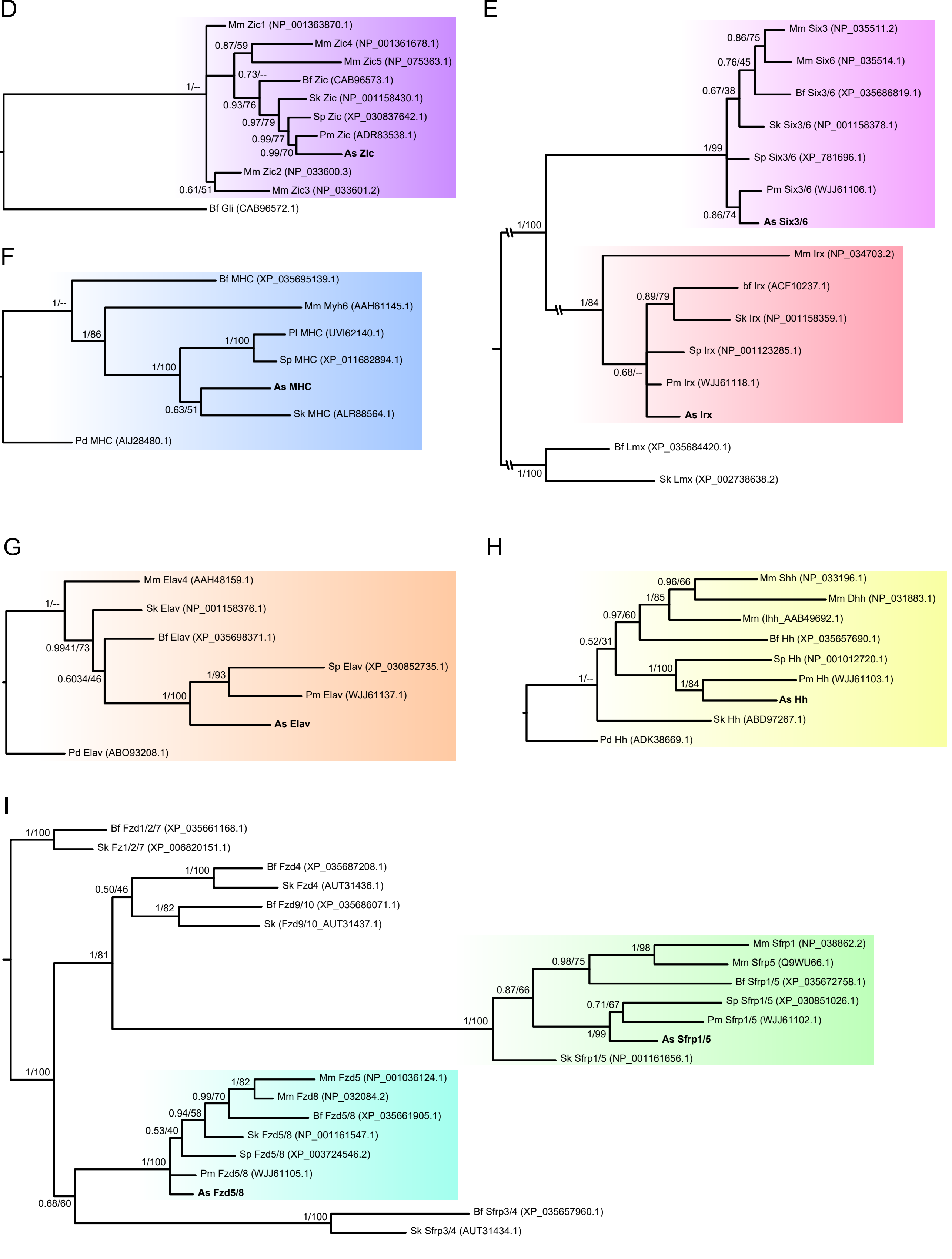

D
Mm Zic1 (NP_001363870.1)
0.87/59
Mm Zic4 (NP_001361678.1)
Mm Zic5 (NP_075363.1)
0.73/--
Bf Zic (CAB96573.1)
1/--
Sk Zic (NP_001158430.1)
0.93/76
Sp Zic (XP_030837642.1)
0.97/79
Pm Zic (ADR83538.1)
0.99/77
0.99/70
As Zic
Mm Zic2 (NP_033600.3)
0.61/51
Mm Zic3 (NP_033601.2)
Bf Gli (CAB96572.1)
E
0.86/75
Mm Six3 (NP_035511.2)
0.76/45
Mm Six6 (NP_035514.1)
0.67/38
Bf Six3/6 (XP_035686819.1)
Sk Six3/6 (NP_001158378.1)
1/99
Sp Six3/6 (XP_781696.1)
Pm Six3/6 (WJJ61106.1)
0.86/74
As Six3/6
1/100
Mm Irx (NP_034703.2)
bf Irx (ACF10237.1)
0.89/79
1/84
Sk Irx (NP_001158359.1)
Sp Irx (NP_001123285.1)
0.68/--
Pm Irx (WJJ61118.1)
As Irx
Bf Lmx (XP_035684420.1)
1/100
Sk Lmx (XP_002738638.2)
F
Bf MHC (XP_035695139.1)
1/--
Mm Myh6 (AAH61145.1)
1/86
Pl MHC (UVI62140.1)
1/100
Sp MHC (XP_011682894.1)
1/100
As MHC
0.63/51
Sk MHC (ALR88564.1)
Pd MHC (AIJ28480.1)
G
Mm Elav4 (AAH48159.1)
1/--
Sk Elav (NP_001158376.1)
Bf Elav (XP_035698371.1)
0.9941/73
0.6034/46
Sp Elav (XP_030852735.1)
1/93
Pm Elav (WJJ61137.1)
1/100
As Elav
Pd Elav (ABO93208.1)
H
0.96/66
Mm Shh (NP_033196.1)
1/85
Mm Dhh (NP_031883.1)
0.97/60
Mm (Ihh_AAB49692.1)
0.52/31
Bf Hh (XP_035657690.1)
Sp Hh (NP_001012720.1)
1/100
1/--
Pm Hh (WJJ61103.1)
1/84
As Hh
Sk Hh (ABD97267.1)
Pd Hh (ADK38669.1)
I
1/100
Bf Fzd1/2/7 (XP_035661168.1)
Sk Fz1/2/7 (XP_006820151.1)
1/100
Bf Fzd4 (XP_035687208.1)
0.50/46
Sk Fzd4 (AUT31436.1)
1/82
Bf Fzd9/10 (XP_035686071.1)
Sk (Fzd9/10_AUT31437.1)
1/81
1/98
Mm Sfrp1 (NP_038862.2)
0.98/75
Mm Sfrp5 (Q9WU66.1)
Bf Sfrp1/5 (XP_035672758.1)
0.87/66
0.71/67
Sp Sfrp1/5 (XP_030851026.1)
1/100
Pm Sfrp1/5 (WJJ61102.1)
1/99
As Sfrp1/5
Sk Sfrp1/5 (NP_001161656.1)
1/100
1/82
Mm Fzd5 (NP_001036124.1)
0.99/70
Mm Fzd8 (NP_032084.2)
0.94/58
Bf Fzd5/8 (XP_035661905.1)
0.53/40
Sk Fzd5/8 (NP_001161547.1)
Sp Fzd5/8 (XP_003724546.2)
1/100
Pm Fzd5/8 (WJJ61105.1)
As Fzd5/8
0.68/60
Bf Sfrp3/4 (XP_035657960.1)
1/100
Sk Sfrp3/4 (AUT31434.1)
