# Supplementary material for: Antero-posterior patterning in the brittle star Amphipholis squamata and the evolution of echinoderm body plans
Source: EvoDevo. 2025 May 31;16:7. doi: 10.1186/s13227-025-00244-8 (PMC12126913; doi:10.1186/s13227-025-00244-8)
Supplement: Supplementary file 9 — Additional file 9: Table 1. Accession numbers of genes of interest [file 13227_2025_244_MOESM9_ESM.docx]

### Supplementary table 1: Accession numbers of genes of interest

| **Gene** | **GenBank accession number** |
| --- | --- |
| *barH* | PV013495 |
| *dmbx* | PV013496 |
| *elav* | PV013497 |
| *fzd5/8* | PV013498 |
| *gbx* | PV013499 |
| *hedgehog* | PV013500 |
| *hox1* | PV013501 |
| *hox2* | PV013502 |
| *hox3* | PV013503 |
| *hox4* | PV013504 |
| *hox5* | PV013505 |
| *hox7* | PV013506 |
| *hox8* | PV013507 |
| *hox9/10* | PV013508 |
| *hox11/13b* | PV013509 |
| *irx* | PV013510 |
| *MHC* | PV013511 |
| *nkx2.1* | PV013512 |
| *otx* | PV013513 |
| *pax2/5/8* | PV013514 |
| *pax6* | PV013515 |
| *sfrp1/5* | PV013516 |
| *six3/6* | PV013517 |
| *zic* | PV013518 |
